# The selective butyrylcholinesterase inhibitor UW‐MD‐95 shows symptomatic and neuroprotective effects in a pharmacological mouse model of Alzheimer's disease

**DOI:** 10.1111/cns.14814

**Published:** 2024-06-17

**Authors:** Allison Carles, Matthias Hoffmann, Matthias Scheiner, Lucie Crouzier, Christelle Bertrand‐Gaday, Arnaud Chatonnet, Michael Decker, Tangui Maurice

**Affiliations:** ^1^ MMDN, Univ Montpellier, INSERM, EPHE Montpellier France; ^2^ Pharmazeutische und Medizinische Chemie Institut für Pharmazie und Lebensmittelchemie, Julius‐Maximilians‐Universität Würzburg Würzburg Germany; ^3^ DMEM, Univ Montpellier, INRAE Montpellier France

**Keywords:** Alzheimer's disease, Aβ_25‐35_, Butyrylcholinesterase, irreversible inhibition, neuroprotection, symptomatic effect, UW‐MD‐95

## Abstract

**Aims:**

Alzheimer's disease (AD) is a devastating dementia characterized by extracellular amyloid‐β (Aβ) protein aggregates and intracellular tau protein deposition. Clinically available drugs mainly target acetylcholinesterase (AChE) and indirectly sustain cholinergic neuronal tonus. Butyrylcholinesterase (BChE) also controls acetylcholine (ACh) turnover and is involved in the formation of Aß aggregates and senile plaques. UW‐MD‐95 is a novel carbamate‐based compound acting as a potent pseudo‐irreversible BChE inhibitor, with high selectivity versus AChE, and showing promising protective potentials in AD.

**Methods:**

We characterized the neuroprotective activity of UW‐MD‐95 in mice treated intracerebroventricularly with oligomerized Aβ_25‐35_ peptide using behavioral, biochemical, and immunohistochemical approaches.

**Results:**

When injected acutely 30 min before the behavioral tests (spontaneous alternation in the Y‐maze, object recognition, or passive avoidance), UW‐MD‐95 (0.3‐3 mg/kg) showed anti‐amnesic effects in Aβ_25‐35_‐treated mice. When injected once a day over 7 days, it prevented Aβ_25‐35_‐induced memory deficits. This effect was lost in BChE knockout mice. Moreover, the compound prevented Aβ_25‐35_‐induced oxidative stress (assessed by lipid peroxidation or cytochrome c release), neuroinflammation (IL‐6 and TNFα levels or GFAP and IBA1 immunoreactivity) in the hippocampus and cortex, and apoptosis (Bax level). Moreover, UW‐MD‐95 significantly reduced the increase in soluble Aβ_1‐42_ level in the hippocampus induced by Aβ_25‐35_.

**Conclusion:**

UW‐MD‐95 appeared as a potent neuroprotective compound in the Aβ_25‐35_ model of AD, with potentially an impact on Aβ_1‐42_ accumulation that could suggest a novel mechanism of neuroprotection.

## Graphical abstract

The symptomatic and neuroprotective activities of the selective BChE inhibitor UW‐MD‐95 were analyzed in the pharmacological mouse model of Alzheimer's disease induced by ICV injection of oligomerized Aβ25‐35. Behavioral, biochemical, and immunofluorescence analyses showed the efficacy of targeting selectively BChE in Alzheimer's disease.

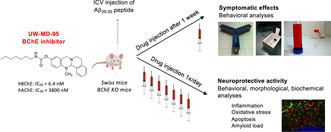

## 1 INTRODUCTION

Alzheimer's disease (AD) is a progressive neurodegenerative disease that causes devastating cognitive and autonomy impairments and ultimately leads to dementia.^1^ The incidence of dementia increases with age and, although the economic burden from dementia in developed countries is rising, neither a curative nor disease‐modifying treatment is yet available. Symptomatic treatments include three cholinesterase inhibitors and an uncompetitive antagonist of the N‐methyl‐D‐aspartate type of glutamate receptors.^2^ The AD brain exhibits characteristic pathological changes that are accumulation of amyloid‐β (Aβ) deposits and tau neurofibrillary tangles (NFTs), neuroinflammation, and a widespread neuronal loss, particularly affecting cholinergic neurons.^3^ Since the underlying causes that initiate and propagate AD remain unclear, clinical trials with novel drugs that target various putative etiopathogenic mechanisms have failed and only passive immunization medicine has recently been authorized for clinical use. Neuroinflammation has long been considered an unspecific consequence of Aβ and NFT formations but is now seen as a main driver in AD pathology.^4^ Therefore, key molecular targets and drivers of neuroinflammation, their interplay, and selective pharmacological inhibitory agents could be a track to design a novel innovative and effective disease‐modifying therapy.

Among the cholinesterases, butyrylcholinesterase (BChE), in addition to acetylcholinesterase (AChE), can also hydrolyze acetylcholine (ACh), and a significant association between lower levels of BChE activity in the gray matter and slower rate of cognitive decline was observed.^5^ BChE is a serine hydrolase with a portfolio of functions in health and disease.^6^ The mechanisms by which BChE affects AD remain poorly understood but go beyond its acute actions. It metabolizes several structurally diverse molecules including xenobiotics and various neuropeptides. BChE has been linked to AD since its activity is increased in AD patient brains^5^ and it appears highly associated with Aβ plaques, NFTs, and cerebral amyloid angiopathy.^7^ While AChE levels in the AD brain are significantly reduced, BChE levels are increased, particularly in the entorhinal and inferotemporal cortex,^5^, ^8^ leading to the suggestion that increase in BChE expression particularly in the orbitofrontal cortex could be used as a sensitive and specific biomarker for AD.^7^ BChE is densely expressed in “malignant” fibrillar Aβ plaques characteristic of AD but not in “benign” non‐fibrillar plaques, which are also found in individuals without dementia.^7^, ^8^ BChE may, therefore, be involved in plaque maturation.^9^ By comparing the 5XFAD mouse strain that develops Aβ plaques and produces BChE and a derived 5XFAD/BChE KO strain that did not produce BChE, Reid & Darvesh^10^ observed that 5XFAD/BChE KO mice have a lower density of Aβ plaques in the cortex. Finally, BChE activity was present in activated microglia associated with lesions in multiple sclerosis^11^ and BChE inhibition resulted in attenuation of inflammation in AD models or in LPS‐induced microglial activation.^12^ BChE activity is, thus, involved in morphological changes associated with toxicity in inflammatory cells around plaques.

We previously reported that genetic invalidation of BChE lowered vulnerability to Aβ toxicity, since learning deficits and oxidative stress observed after intracerebroventricular (ICV) injection of oligomeric Aβ_25‐35_ peptide were attenuated in BChE KO mice.^13^ We described new carbamate‐based chemical scaffolds that act as highly selective and potent pseudo‐irreversible BChE inhibitors, with nanomolar potencies and high efficacies in vivo.^14^ One compound, namely, 13‐methyl‐5,8,13,13a‐tetrahydro‐6*H*‐isoquinolino[1,2‐*b*]quinazolin‐10‐yl heptylcarbamate (UW‐MD‐95), prevented Aβ_25‐35_‐induced memory deficits and neuroinflammation in mice. Chronic administration of the BChE inhibitor showed protection even after an in vivo wash‐out period, suggesting disease‐modifying effects. In contrast, AChE inhibitors have a purely acute effect.^15^ Moreover, drugs with longer duration of inhibition in vitro, due to slower off‐hydrolysis, appeared more active in vivo.^14^

In the present study, we characterize that at the behavioral, biochemical, and morphological levels, the symptomatic and neuroprotective activities of UW‐MD‐95 in the mouse model of AD induced by ICV injection of Aβ_25‐35_ peptide. One week after administration of Aβ_25‐35_, mice developed oxidative stress, neuroinflammation, apoptosis, and learning deficits.^16^, ^17^ UW‐MD‐95 (0.3‐3 mg/kg) was administered intraperitoneally either 7 days after Aβ_25‐35_, to examine the symptomatic effects or once a day during 1 week after Aβ_25‐35_, to examine the neuroprotective effects.^16^, ^17^ Memory abilities were analyzed using different behavioral tests and neuroprotection was examined in the hippocampus or cortex using biochemical analyses targeting oxidative stress, neuroinflammation, apoptosis markers, and amyloid species content, as well as using immunohistochemical and histological analyses of the mouse brains.

## 2 MATERIALS AND METHODS

### 2.1 Animals

Male Swiss CD‐1 (RjOrl:SWISS) mice, aged 7‐9 weeks, were supplied by Janvier (Le Genest‐Saint‐Isle, France) and housed at the animal facility of the University of Montpellier (agreement # D34‐172‐23). Homozygous BuChE KO founders were provided by Dr. O. Lockridge (Eppley Institute, University of Nebraska Medical Center, Omaha, NE, USA).^18^ The colony was then maintained on a pure 129/Sv strain at the animal facility of INRAE in Montpellier (agreement no. D34‐172‐10). Wild‐type (WT) and homozygous (KO) littermates were transferred to the animal facility of the University of Montpellier (CECEMA, agreement # D34‐172‐23) at the age of 3 months, at least 1 week before the behavioral experiments start. Mice were housed in a regulated environment (23 ± 1°C, 50% humidity, 12‐h light/dark cycle), in groups (8‐10 individuals), with free access to food and water. Animal procedures followed the European Union Directive 2010/63 and the ARRIVE guidelines. They were authorized by the National Ethic Committee (Paris).

### 2.2 Drug and peptides

**FIGURE 1:**
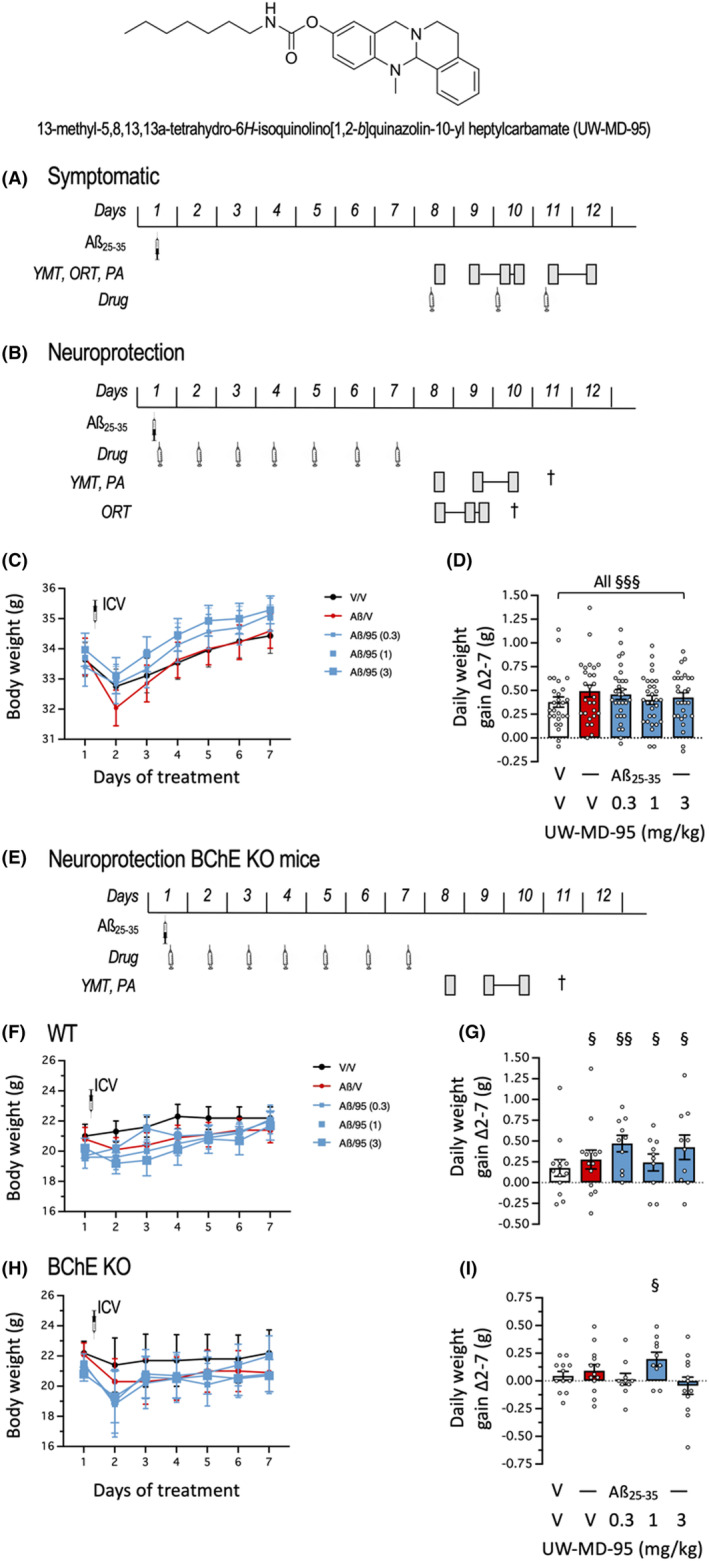
Chemical structure of UW‐MD‐95 and experimental protocols for (A) symptomatic or (B) neuroprotection experiments in Swiss mice, and (E) neuroprotection experiments in BChE KO mice. On day 1, the Aβ_25‐35_ peptide was administered ICV. In (A), mice were tested symptomatically and injected with UW‐MD‐95 after 1 week, 60 min before testing as indicated. In (B, E), UW‐MD‐95 was administered IP o.d., and mice were tested after 1 week without drug treatment. Measures of daily body weight and daily weight gain in: (C, D) Swiss, (F, G) wild‐type (WT) and (H, I) BChE KO mice treated o.d. with UW‐MD‐95. YMT, Y‐maze test; ORT, object recognition test; PA, step‐through passive avoidance; †, euthanasia for brain dissection; V, vehicle solution; 95, UW‐MD‐95; Aß, Aβ_25‐35_. ^§^ *p* < 0.05, ^§§^ *p* < 0.01, ^§§§^ *p* < 0.0001 versus 0 level, one‐sample *t*‐test in (D, G, I).

13‐methyl‐5,8,13,13a‐tetrahydro‐*6H*‐isoquinolino[1,2‐*b*]quinazolin‐10‐yl heptylcarbamate (UW‐MD‐95) (Figure 1) was synthetized in the laboratory (Würzburg), as described.^19^ It was solubilized in dimethyl sulfoxide/physiological saline (NaCl 0.9%) with a final ratio of 60/40 (vehicle solution). Scopolamine hydrobromide was purchased from Sigma Aldrich (St Quentin‐Fallavier, France) and solubilized in physiological saline. The compounds were administered intraperitoneally (IP) or subcutaneously (SC), as precised, in a volume of 100 μL per 20‐g body weight.

Amyloid‐β^25^, ^26^, ^27^, ^28^, ^29^, ^30^, ^31^, ^32^, ^33^, ^34^, ^35^ (Aβ_25‐35_) peptide was from Eurogentec (Angers, France). It was solubilized in distilled water at 3 mg/mL and stored at −20°C until further use. Before injection, the peptide was oligomerized by incubation at 37°C for 4 days. Control injection was performed with vehicle solution (distilled water), as we previously described no effect of antisense or control peptide. Intracerebroventricularly (ICV) injections were performed as described.^20^

### 2.3 Experimental series

We examined two compound administration procedures. First, symptomatic effects were analyzed in Aβ_25‐35_‐treated mice by injecting the compounds just before the behavioral tests. Aβ_25‐35_ was injected ICV on day 1 and the drug was injected after 7 days, 30 min before the behavioral tests: spontaneous alternation, session 2 of the object recognition test, or passive avoidance training (as illustrated in Figure 1A). Second, the neuroprotective effects were analyzed by repeatedly injecting the mice for 1 week starting on the day of peptide injection. The drug was injected o.d. from day 1 to day 7 (Figure 1B) and mice were tested for spontaneous alternation and passive avoidance or object recognition, without further compound injection. They were euthanized on day 10 or 11 for immunofluorescence chemistry or biochemical assays. Body weights were measured o.d. before each compound injection and analyzed (Figure 1C,D). An additional series included BChE KO mice that were injected o.d. from day 1, after the peptide injection, to day 7 and tested for spontaneous alternation and passive avoidance in series (Figure 1E). Body weight was measured o.d. before each drug injection and analyzed (Figure 1F–I).

### 2.4 Behavioral tests

#### 2.4.1 Spontaneous alternation in the Y‐maze

The Y‐maze is made of gray polyvinylchloride. Each arm is 40 cm long, 13 cm high, 3 cm wide at the bottom, 10 cm wide at the top, and converging at an equal angle. Each mouse was placed at the end of one arm and moved freely through the maze during 8 min. The series of arm entries, including possible returns into the same arm, were checked visually. An alternation was defined as consecutive entries into all three arms. The number of maximum alternations was, therefore, the total number of arm entries minus two and the percentage of alternation was calculated as (actual alternations/maximum alternations) × 100.^16^, ^20^ Exclusion criteria were set: locomotion <10 or percentage of alternation <25% or >90%. Animals showing these criteria were excluded from the calculations. Attrition was 5% in this study.

#### 2.4.2 Step‐through passive avoidance

A two‐compartment box measuring 15 × 20 × 15 cm high, each, one with white polyvinylchloride walls and the other with black polyvinylchloride walls and with a grid floor. A guillotine door separated the compartments. A 60 W lamp placed 40 cm above the box lighted up the white compartment during the experiment. Scrambled footshocks (0.3 mA for 3 s) could be delivered to the grid using a shock generator (Lafayette Instruments, Lafayette, USA). The guillotine door was initially closed during the training session. Each mouse was placed in the white compartment and the door was raised after 5 s. When the mouse entered the darkened compartment and placed its four paws on the grid, the door was closed and the footshocks were delivered. The step‐through latency (STL‐Tg) spent to enter the dark compartment and the number of vocalizations were recorded. The retention test was performed after 24 h. Each mouse was placed again in the white compartment and the door was raised after 5 s. The step‐through latency (STL‐R) was recorded up to 300 s.^16^, ^17^

#### 2.4.3 Object recognition test

In session 1, animals were placed in a square 50 × 50 cm^2^ arena for 10 min. In session 2, after 24 h, two similar objects were placed at ¼ and ¾ of a diagonal of the arena. The mouse activity and nose position were recorded for 10 min (Nosetrack® software, Viewpoint, Lissieu, France). The number of contacts and duration of contacts with the objects were measured. In session 3, after 1 h, the object in position 2 was replaced by a novel one with different shape, color, and texture. The animal activity was recorded for 10 min and analyzed. A preferential exploration index was calculated as the ratio of the number (or duration) of contacts with the object in position 2 over the total number (or duration) of contacts with the two objects. Animals showing no contact with one object or less than 10 contacts with objects during session 2 or 3 were discarded from the study. Attrition was 9% in this study.

### 2.5 Lipid peroxidation measures

Mice were euthanized by decapitation 11 days after Aβ_25‐35_ injection and brains were dissected out, the hippocampi weighed, frozen in liquid nitrogen, and stored at −80°C. The level of lipid peroxidation was determined as previously described.^16^

### 2.6 Cytochrome c release

The mouse hippocampi were homogenized in ice‐cold homogenization buffer (250 μM sucrose, 10 mM HEPES, pH 7.4), with a protease and phosphatase inhibitor cocktail (Roche Diagnostics, Meylan, France) in a final volume of 250 μL. Homogenates were centrifuged (600 ***g***, 5 min) and the supernatant was collected and centrifuged again (10,300 ***g***, 20 min). The second supernatant corresponding to the cytosolic fraction and the pellet corresponding to the crude mitochondrial fraction were separated. The mitochondrial fraction was resuspended in 50 μL of ice‐cold isolation buffer (250 mM mannitol, 5 mM HEPES, 0.5 mM EGTA, pH 7.4). Protein concentration was determined using a BCA assay (Pierce Biotechnology, Rockford, IL, USA). Proteins (20 μg) were resolved on a 12% SDS‐polyacrylamide gel and transferred on a polyvinylidene fluoride (PVDF) membrane (GE Healthcare, Orsay, France). After 1 h blocking in 5% non‐fat dry milk in a 20 mM Tris‐buffered saline pH 7.5 buffer with 0.1% Tween‐20 (TBS‐T), membranes were incubated overnight at 4°C with primary antibodies: mouse anti‐cytochrome c (CytC, dilution 1/1000; BioLegend, San Diego, CA, USA), or mouse anti‐oxphos‐complex IV subunit I (Oxphos, 1/1000; Invitrogen Life Technologies, St Aubin, France). After brief washes, membranes were incubated for 1 h at room temperature with the secondary antibody, goat anti‐mouse IgG peroxidase conjugate (1/2000; Sigma‐Aldrich). The immunoreactive bands were visualized with the enhanced chemiluminescence reagent (ECL, Millipore, Molsheim, France) using an Odyssey® Fc fluorescent imaging system (Li‐Cor, Eurobio, Courtaboeuf, France). The intensity of peroxidase activity was quantified using the Odyssey® Fc software (Li‐Cor).

### 2.7 Enzyme‐linked immunosorbent assays (ELISA)

The mouse hippocampi were homogenized after thawing in 1 mL of fresh lysis buffer (3 IS007, Cloud‐Clone) and sonicated on ice for 2 × 10 s. After centrifugation (10,000 ***g***, 5 min, 4°C), supernatants were aliquoted and stocked at −80°C. Elisa assays were used to monitor protein levels in tumor necrosis factor‐α (TNFα), interleukin‐6 (IL‐6), allograft inflammatory factor‐1 (IBA‐1), glial fibrillary acidic protein (GFAP), B‐cell lymphoma 2 (Bcl‐2), Bcl‐2–associated X (Bax), Aβ_1‐42_, and Aβ_1‐40_ (see Table S1 for kit references). For each assay, absorbance was read at 450 nm and sample concentration was calculated using the standard curve. Results are expressed in ng of marker per mg of protein and in % of the control (V + V) value.

### 2.8 Brain fixation and slicing

Eleven days after Aβ_25‐35_ injection, mice were anesthetized with 200 μL of a premix of ketamine (80 mg/kg) and xylazine (10 mg/kg) injected IP and transcardially perfused with 50 mL of saline solution followed by 50 mL of Antigenfix® (Diapath). The brains were kept for 48 h post‐fixation in Antigenfix solution, at 4°C. They were then placed in a sucrose 30% phosphate buffer saline (PBS) solution. Serial coronal frozen sections (25 μm thickness) were cut to include the cortex and hippocampal formation, between Bregma +1.80 and −2.80 according to Paxinos & Franklin,^21^ with a freezing microtome (Microm HM 450, ThermoFisher), collected in 24‐well plates, stored in cryoprotectant at −20°C, and then mounted on slides.

### 2.9 Immunohistochemical labeling of microglia (IBA‐1) and astrocytes (GFAP)

For immunohistochemical labeling, slices in 24‐well plate were incubated overnight at +4°C with Rabbit polyclonal anti‐IBA‐1 (1:250, 019‐19741, Wako) and mouse monoclonal anti‐GFAP (1:400, G3893, Sigma‐Aldrich). Then, slices were incubated 1 h at room temperature with secondary Cy3 affinipure donkey anti‐rabbit IgG(H + L) (1:1000, 711‐165‐152, Jackson Immunoresearch) and secondary Alexa Fluor 488 AffiniPure donkey anti‐rabbit IgG(H + L) (1:1000, 711‐545‐152, Jackson Immunoresearch) antibodies. Slices were incubated for 5 min with DAPI 10 μg/mL (1:50,000) and rinsed with potassium phosphate buffer saline. Finally, slices were mounted with Dako Fluorescence Mounting Medium (Dako). Pictures were taken with a fluorescent Microscope (Zeiss).

### 2.10 Statistical analyses

Analyses were done using Prism v10.0 software (GraphPad, San Diego, CA, USA). Data were analyzed using one‐way analyses of variance (anova, *F* value) or two‐way anova with the ICV Aβ_25‐35_ treatment and IP drug treatment as independent factors, followed by a Dunnett's or Mann‐Whitney's test. Normality was analyzed using Bartlett's test. Passive avoidance latencies were expressed as median and interquartile range and represented as box‐and‐whiskers. They were non‐parametric data as upper cut‐off time were set and were analyzed using a non‐parametric Kruskal‐Wallis anova (*H* value). *Post‐hoc* comparisons were done using a Dunn's test. Daily weight gains and object preferences were analyzed using a one‐sample *t* test versus the chance level (0% or 50%). Significance levels were *p* < 0.05, *p* < 0.01, and *p* < 0.001. Statistical data are detailed in Table S2.

## 3 RESULTS

As the vehicle solution used for UW‐MD‐95 contained a high percentage of DMSO, we carefully checked weight gain profiles of the mice during the repeated compound treatment (Figure 1C,D,F‐I). Generally, Swiss mice lost 1‐g body weight after the surgery (ICV injection of vehicle solution or Aβ_25‐35_ peptide) but then gradually recovered a regular weight gain (Figure 1C). The daily weight gain was significantly positive and similar among treatment groups (Figure 1D). For the additional series including BChE KO mice, body weights were also measured o.d. and profiles showed that WT or BChE KO animals, with a 129S/v background strain, showed only a slight weight loss after ICV injection and a slight weight gain during the week of treatment (Figure 1F,H). The daily weight gain was positive for WT mice (Figure 1G) and close to zero for BChE KO mice (Figure 1I), but remarkably sustained in the groups treated with UW‐MD‐95. We, therefore, concluded that for both mouse strains, the drug and vehicle injections were very well tolerated.

### 3.1 Anti‐amnesic effects of the selective BChE inhibitor UW‐MD‐95 in Aβ_25‐35_‐injected mice

**FIGURE 2:**
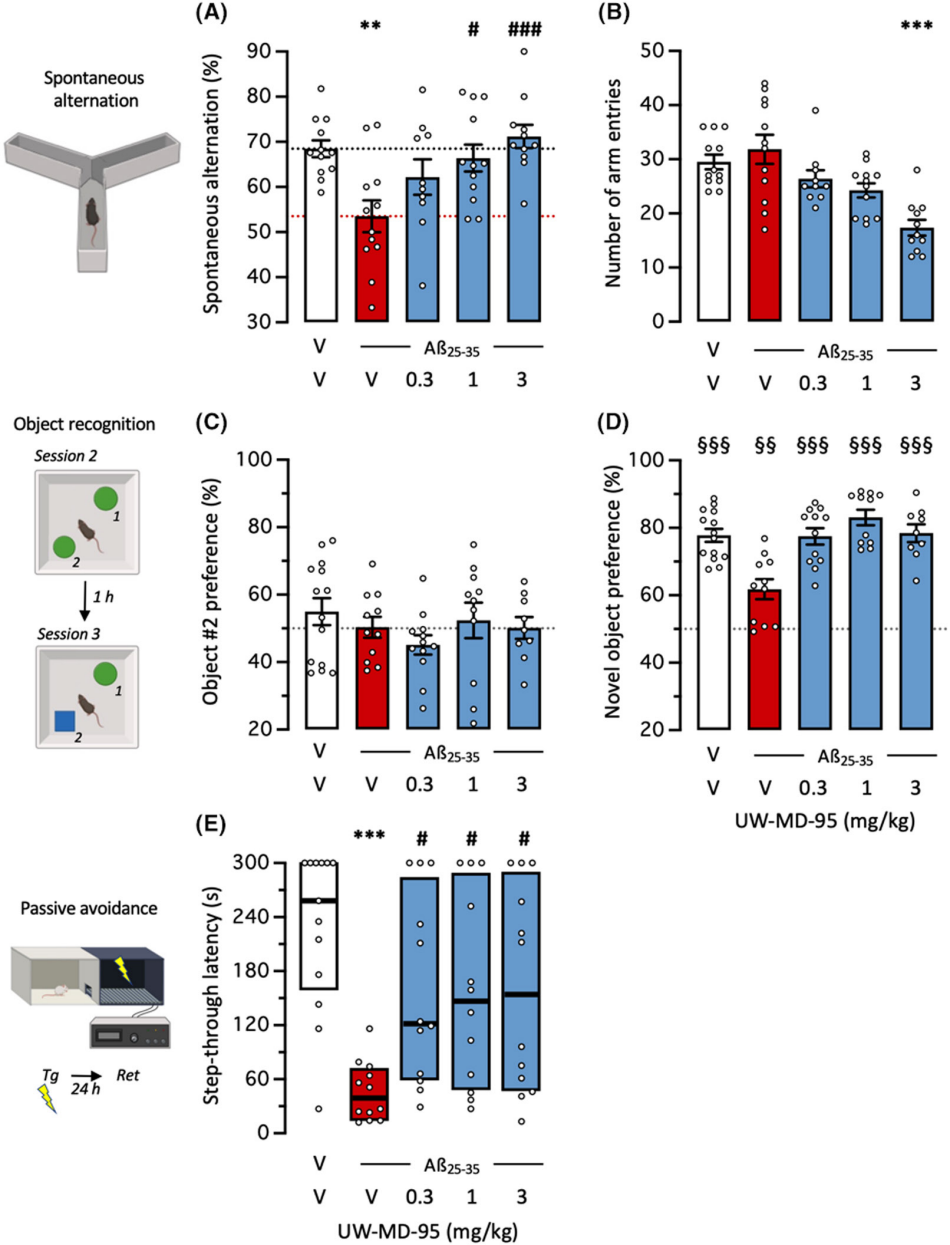
Symptomatic effect of UW‐MD‐95 on Aβ_25‐35_‐induced learning impairments in mice: (A) spontaneous alternation performance and (B) total number of arm entries in the Y‐maze test; (C) Session 2 (same objects) and (D) session 3 (with a novel object) in the object recognition test; (E) passive avoidance retention session (Tg, training session; Ret, retention session). Animals received UW‐MD‐95 (0.3–3 mg/kg IP) 60 min before the Y‐maze test session, session 2 of the object recognition test, or the passive avoidance training session. For the object recognition test, exploration preferences are calculated using durations of contact in session 2 with the object in position 2 (C) and in session 3 with the novel object, placed in position 2 (D). Data show mean ± SEM in (A–D) and median and interquartile range in (E). **p < 0.01, ***p < 0.001 versus (V + V)‐treated group; #*p* < 0.05, ###*p* < 0.001 versus (V + Aβ_25‐35_)‐treated group; Dunnett's test in (A, B), Dunn's test in (E). ^§§^ *p* < 0.01, ^§§§^ *p* < 0.001 versus 50% level, one‐sample *t*‐test in (D).

We first analyzed the symptomatic effects of UW‐MD‐95 on Aβ_25‐35_‐induced learning deficits in mice. The drug was injected in the 0.3–3 mg/kg dose range, 30 min before the Y‐maze test or before the training session in long‐term memory tests, and 7 days after Aβ_25‐35_ injection (Figure 1A). UW‐MD‐95 dose dependently attenuated Aβ_25‐35_‐induced spontaneous alternation deficits in mice with significant effects at 1 and 3 mg/kg (Figure 2A). The drug dose dependently decreased the number of arms explored during the 8‐min session, with a significant effect at the highest dose tested (Figure 2B) that suggested some impairing effect on locomotion or exploration at high doses. In the object recognition test, none of the treatments affected the balanced exploration of the two similar objects (Figure 2C), while during the last session, Aβ_25‐35_ decreased the novel object exploration from 80% to 60% (Figure 2D). UW‐MD‐95 attenuated the Aβ_25‐35_‐induced decrease at all doses tested (Figure 2D). In the passive avoidance test, UW‐MD‐95 attenuated Aβ_25‐35_‐induced a deficit in step‐through latency at all doses tested (Figure 2E).

In a supplementary experiment, we tested the effect of UW‐MD‐95 against the learning deficit induced by the muscarinic ACh receptor antagonist scopolamine (Figure S1). UW‐MD‐95 dose dependently attenuated scopolamine‐induced spontaneous alternation deficits in mice with significant effects at 0.3 and 1 mg/kg (Figure S1A). The co‐treatment of UW‐MD‐95 + scopolamine significantly increased the number of arm entries during the session (Figure S1B). In the passive avoidance test, UW‐MD‐95 significantly attenuated scopolamine‐induced step‐through latency deficit at the dose of 0.3 mg/kg (Figure S1C).

### 3.2 Protective effects of UW‐MD‐95 against Aβ_25‐35_‐induced memory deficits in mice

**FIGURE 3:**
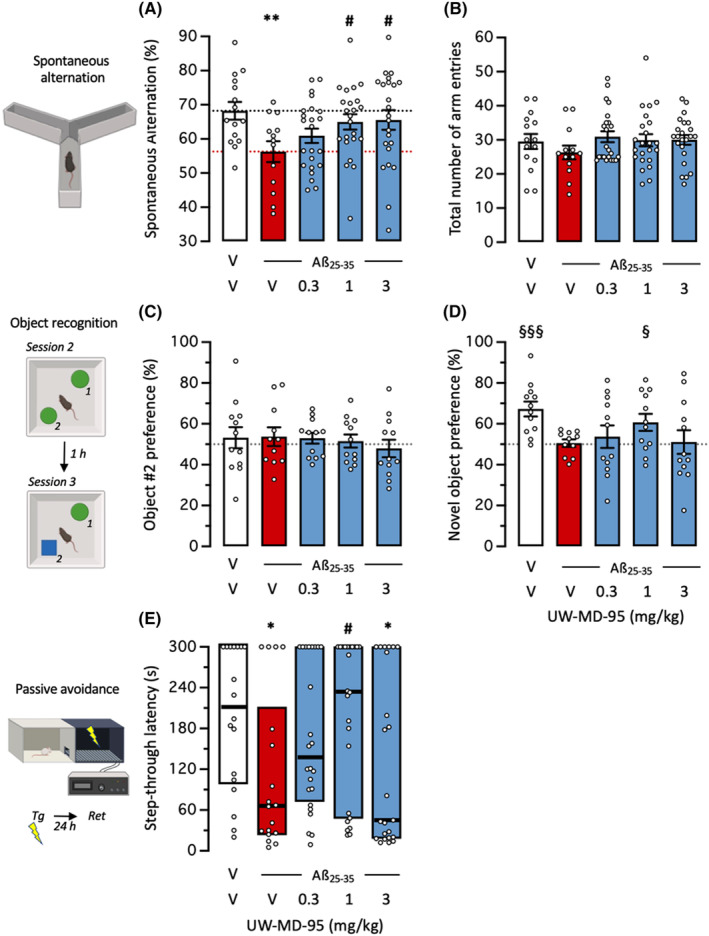
Neuroprotective effect of UW‐MD‐95 on Aβ_25‐35_‐induced learning impairments in mice: (A) spontaneous alternation performance and (B) total number of arm entries in the Y‐maze test; (C) Session 2 (same objects) and (D) session 3 (with a novel object) in the object recognition test; (E) passive avoidance retention session (Tg, training session; Ret, retention session). Animals received UW‐MD‐95 (0.3–3 mg/kg IP) o.d. during 7 days after Aβ_25‐35_ injection. For the object recognition test, exploration preferences are calculated using durations of contact in session 2 with the object in position 2 (C) and in session 3 with the novel object, placed in position 2 (D). Data show mean ± SEM in (A–D) and median and interquartile range in (E). *p < 0.05, **p < 0.01 versus (V + V)‐treated group; #*p* < 0.05 versus (V + Aβ_25‐35_)‐treated group; Dunnett's test in (A), Dunn's test in (E). ^§^ *p* < 0.05, ^§§§^ *p* < 0.001 versus 50% level, one‐sample *t*‐test in (D).

We then analyzed the protective potential of the BChE inhibitor against Aβ_25‐35_‐induced memory deficits and toxicity in mice. UW‐MD‐95 was injected o.d. between day 1 and day 7 after Aβ_25‐35_ and mice were tested for memory without further drug injection (Figure 1B). The compound treatment prevented Aβ_25‐35_‐induced spontaneous alternation deficits in mice (Figure 3A), at the doses of 1 and 3 mg/kg, without affecting the total number of arm entries (Figure 3B). In the object recognition test, the UW‐MD‐95 treatment did not affect the equal exploration of the two similar objects (Figure 3C). During the last session, Aβ_25‐35_ altered the increase in exploration of the novel object, and this deficit was significantly attenuated with the 1 mg/kg treatment of UW‐MD‐95 (Figure 3D). A similar bell‐shaped effect was observed in the passive avoidance test (Figure 3E). UW‐MD‐95 attenuated significantly the Aβ_25‐35_‐induced deficit at the dose of 1 mg/kg but in a bell‐shaped manner (Figure 3E).

**FIGURE 4:**
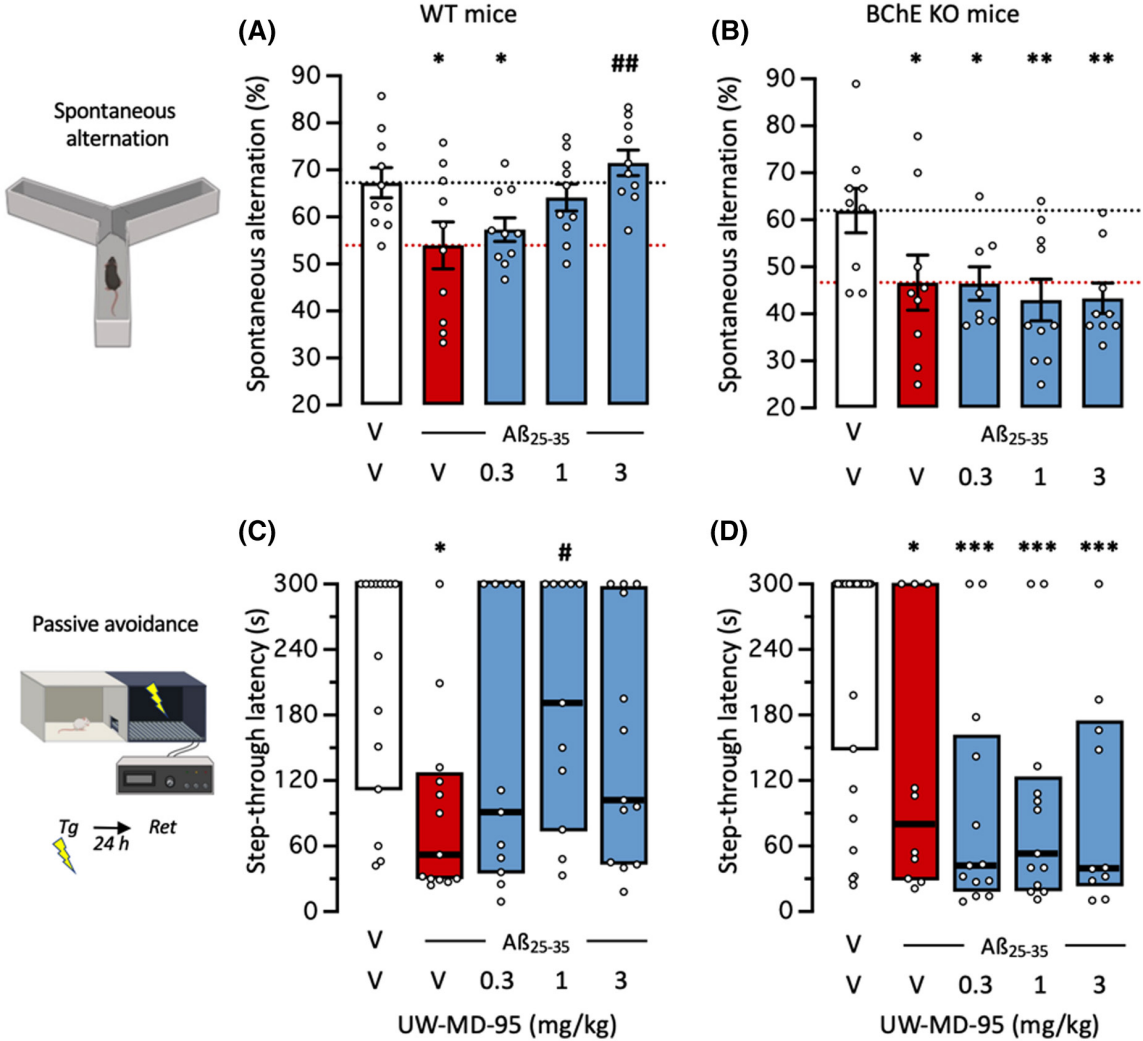
Lack of protective effect of UW‐MD‐95 on Aβ_25‐35_‐induced learning impairments in BChE KO mice: (A, B) spontaneous alternation performance and (C, D) passive avoidance retention for (A, C) WT mice and (B, D) BChE KO mice. Animals received UW‐MD‐95 (0.3–3 mg/kg IP) 60 min before the Y‐maze test session or passive avoidance training session. Data show mean ± SEM in (A, B) and median and interquartile range in (E). *p < 0.05, **p < 0.01, ***p < 0.001 versus (V + V)‐treated group; ^#^ *p* < 0.05, ^##^ *p* < 0.01 versus (V + Aβ_25‐35_)‐treated group; Dunnett's test in (A, B), Dunn's test in (C, D).

In order to ascertain the involvement of the BChE inhibition in the compound effect, UW‐MD‐95 was tested in BChE KO mice, and WT controls, after ICV injection of Aβ_25‐35_ (Figure 4). In WT and BChE KO animals, Aβ_25‐35_ altered spontaneous alternation and the deficit was alleviated by increasing doses of UW‐MD‐95 (Figure 4A) but not in BChE KO mice (Figure 4B). The Aβ_25‐35_‐induced alteration of step‐through latency was also attenuated by UW‐MD‐95 in WT mice, but in a bell‐shaped manner (Figure 4C), and unaltered in BChE KO mice (Figure 4D). The lack of effect of UW‐MD‐95 in BChE KO mice confirmed that inhibition of the enzyme is involved in the drug's neuroprotective effect.

In a series of supplementary experiments, we tested the potentiality of a single UW‐MD‐95 administration, 20 min before the Aβ_25‐35_ injection. One week after the injections, mice were tested for spontaneous alternation and passive avoidance (Figure S2). UW‐MD‐95 dose dependently attenuated Aβ_25‐35_‐induced spontaneous alternation deficits in mice with significant effects at 1 and 3 mg/kg (Figure S2A). The drug treatment also decreased the number of arm entries, with a significant effect at 1 mg/kg (Figure S2B). In the passive avoidance test, Aβ_25‐35_ induced a significant decrease in the step‐through latency that was attenuated by UW‐MD‐95, significantly at the 1 mg/kg dose (Figure S2C).

### 3.3 Protective effects of UW‐MD‐95 against Aβ_25‐35_‐induced toxicity in mice

**FIGURE 5:**
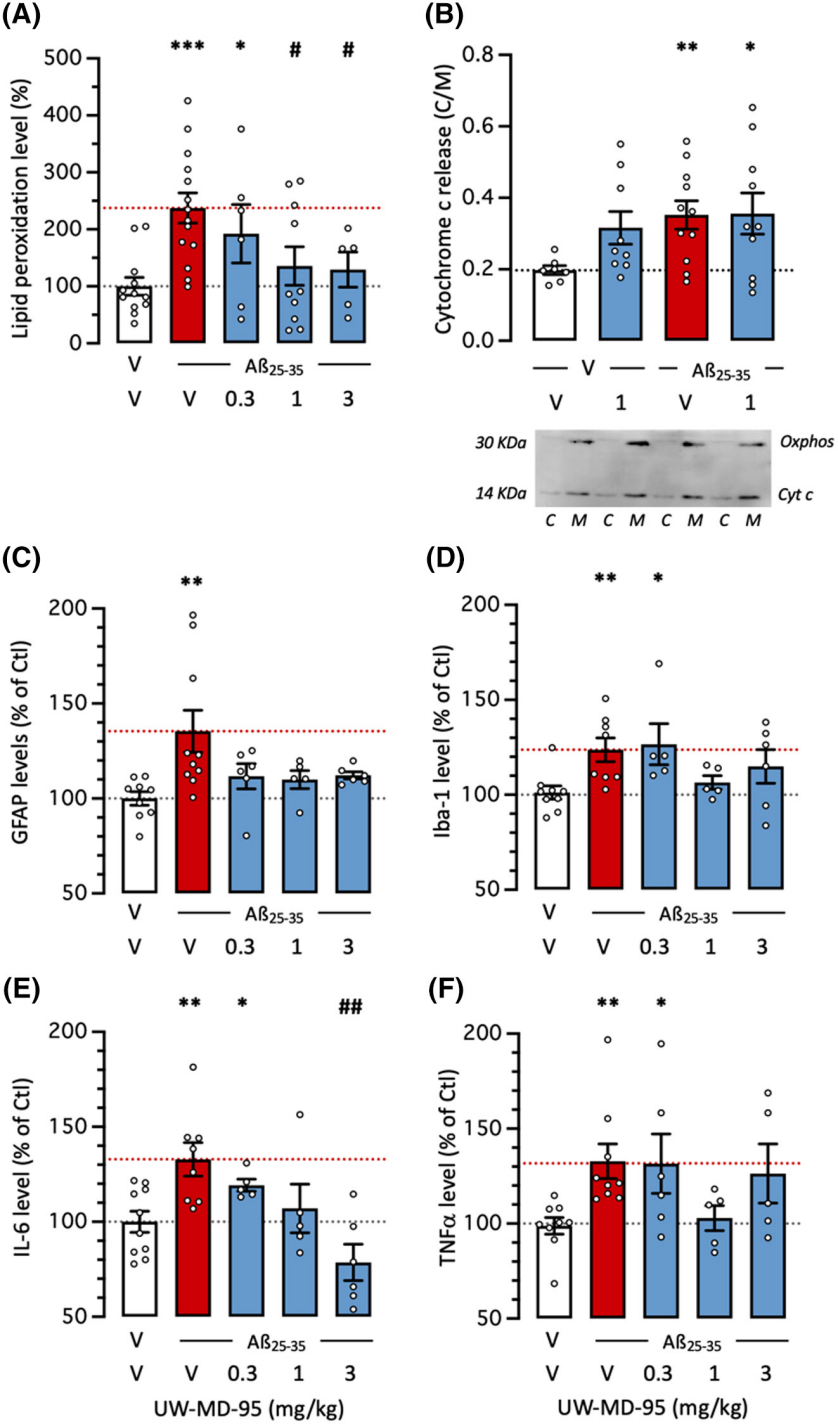
Protective effects of UW‐MD‐95, administered at 0.3–3 mg/kg IP, on Aβ_25‐35_‐induced (A, B) oxidative stress and mitochondrial alteration, (C) GFAP and (D) IBA‐1 glial markers levels, and (E) IL‐6 and (F) TNFα cytokine levels, measured by ELISA in the mouse hippocampus 11 days after Aβ_25‐35_ ICV injection. In (B), the cytochrome c release was analyzed 5 days after Aβ_25‐35_ ICV injection by western blot in mitochondrial (M) and cytosol (C) fractions in cortex extracts. A typical blot showing Oxphos mitochondrial marker and cytochrome c labeling is shown. *p < 0.05, **p < 0.01, ***p < 0.001 versus (V + V)‐treated group; ^#^ *p* < 0.05, ^##^ *p* < 0.01 versus (V + Aβ_25‐35_)‐treated group; Dunnett's test.

Several biochemical parameters of Aβ_25‐35_‐induced toxicity were analyzed in mouse hippocampus. First, oxidative stress was analyzed using the measure of lipid peroxidation (Figure 5A). The Aβ_25‐35_ treatment significantly increased lipid peroxidation level by +153% and UW‐MD‐95 dose dependently prevented this increase with significant effects at 1 and 3 mg/kg (Figure 5A). Alteration of mitochondria integrity was measured using cytochrome c release into the cytosol. Cytosolic and mitochondrial fractions were isolated and the latter was identified using Oxphos immunoreactivity (Figure 5B). Aβ_25‐35_ induced a significant +78% increase in cytochrome c release, measured as cytosol/mitochondria content ratio, that was unaffected by UW‐MD‐95, tested at 1 mg/kg (Figure 5B).

Several markers of neuroinflammation were analyzed in hippocampus extracts. The levels of cellular markers of reactive astrocytes and microglia were significantly increased 2 weeks after Aβ_25‐35_: +35% for GFAP (Figure 5C) and +24% for IBA‐1 (Figure 5D). The UW‐MD‐95 treatment attenuated these increases, at all doses tested for GFAP (Figure 5C) and at 1 and 3 mg/kg for IBA‐1 (Figure 5D). Cytokine contents were increased: +33% for IL‐6 (Figure 5E) and + 34% for TNFα (Figure 5F). The UW‐MD‐95 treatment prevented the increases in these cytokines with a marked effect at the dose of 1 or 3 mg/kg (Figure 5E,F).

**FIGURE 6:**
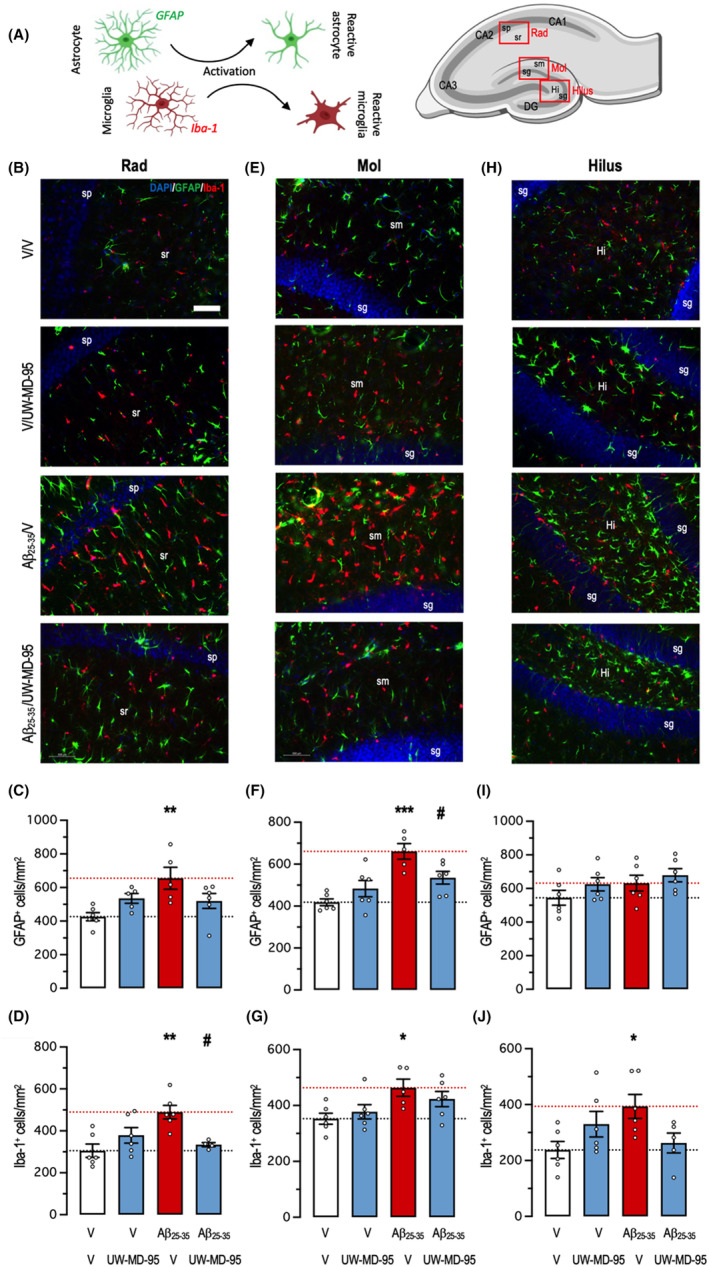
Protective effects of UW‐MD‐95, administered at 1 mg/kg IP, on glial reaction in the hippocampus of Aβ_25‐35_‐treated mice. (A) Astroglial cell were immunolabeled with GFAP, microglial cells were immunolabeled with IBA‐1 and cell bodies with DAPI. Localization of the three areas of interest is shown in the mouse hippocampus. (B–D) stratum radiatum (Rad), (E–G) molecular layer (Mol), and (H‐J) Hilus/polymorph layer of the dentate gyrus. (B, E, H) Typical immunofluorescence micrographs of coronal 25‐μm‐thick sections (blue: DAPI, green: GFAP, red: IBA‐1), (C, F, I) quantifications of GFAP immunolabeling and (D, G, J) quantifications of IBA‐1 immunolabeling. Four to six slices were counted per animal and the value averaged for each area. sp, stratum pyramidale; sr, stratum radiatum; sg, stratum granulare; sm, stratum moleculare; DG, dentate gyrus; Hi, hilus. Scale bar in (B) = 50 μm. *p < 0.05, **p < 0.01, ***p < 0.001, Dunnett's test.

**FIGURE 7:**
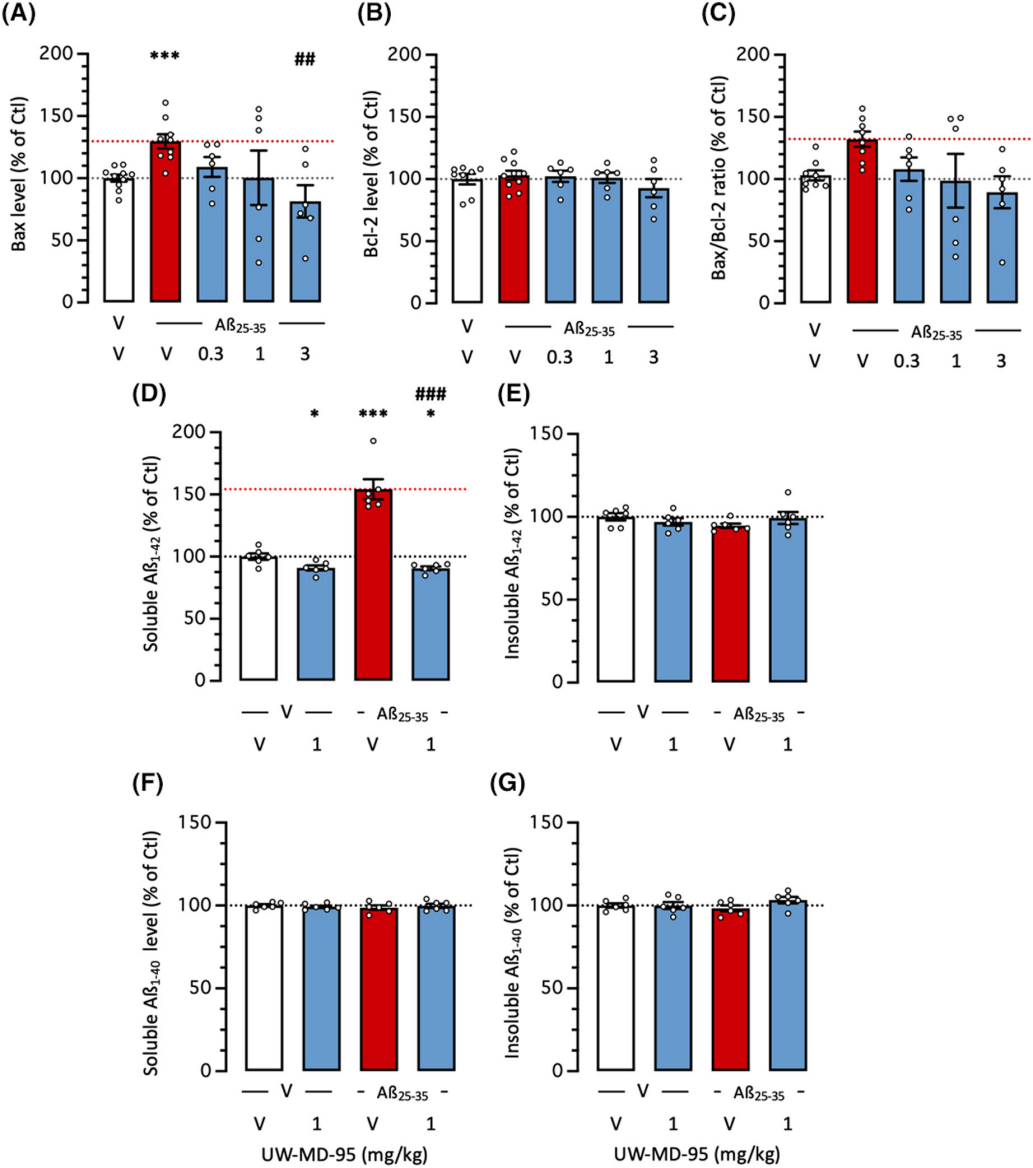
Protective effects of UW‐MD‐95, administered at 0.3–3 mg/kg IP, on Aβ_25‐35_‐induced (A–C) apoptosis markers and (D‐G) amyloid load. (A) Bax level, (B) Bcl‐2 level, and (C) Bax/Bcl‐2 ratio were determined in cortex extracts. Contents in (D) soluble Aβ_1‐42_, (E) insoluble Aβ_1‐42_, (F) soluble Aβ_1‐40_, and (G) insoluble Aβ_1‐40_ were measured by ELISA in cortex 11 days after Aβ_25‐35_ ICV injection. *p < 0.05, **p < 0.01, ***p < 0.001 versus (V + V)‐treated group; ^#^ *p* < 0.05, ^##^ *p* < 0.01, ^###^ *p* < 0.001 versus (V + Aβ_25‐35_)‐treated group; Dunnett's test.

Neuroinflammation was also analyzed using immunohistofluorescence in three distinct areas of the hippocampus: the *stratum radiatum* (Rad), *stratum moleculare* (Mol), and polymorph layer of the dentate gyrus (Hilus) (Figure 6A). GFAP and IBA‐1 immunolabellings in the hippocampal subfields showed intense astroglial and microglial reactions. Cell counting showed significant increases in the Rad (Figure 6B–D) and Mol (Figure 6E–G) and only a trend for GFAP in Hilus (Figure 7H–J). UW‐MD‐95 treatment at 1 mg/kg attenuated the increase in GFAP immunolabeling in the Rad (Figure 6B,C) and Mol (Figure 6E,F) but not in the Hilus (Figure 6H,I).

UW‐MD‐95 treatment decreased Aβ_25‐35_‐induced increases in IBA‐1 labelling in Rad (Figure 6B,D) and Hilus (Figure 6H,J) but less efficiently in Mol (Figure 6E,G).

Aβ_25‐35_ increased the content in pro‐apoptotic protein Bax by +30% (Figure 7A). UW‐MD‐95 significantly prevented this increase at the highest dose tested (Figure 7A). The treatments (Aβ_25‐35_ or UW‐MD‐95) failed to affect the levels of the anti‐apoptotic protein Bcl‐2 (Figure 7B). However, the Bax/Bcl‐2 ratio was increased by Aβ_25‐35_ (+32%) and this increase was dose dependently prevented by the compound (Figure 7C).

The ICV injection of Aβ_25‐35_ did not result in visible amyloid deposits but increased amyloid load by generating Aβ_1‐42_ in the hippocampus and cortex of mice.^22^ We analyzed the soluble and insoluble forms of Aβ_1‐40_ and Aβ_1‐42_ in the mouse cortex that corresponded to small oligomeric aggregates representing the most toxic amyloid species and constituted fibrillar Aβ deposits^23^ (Figure 7D–G). The Aβ_25‐35_ treatment significantly increased by +54% the level of soluble Aβ_1‐42_ and this increase was fully prevented by UW‐MD‐95 tested at 1 mg/kg (Figure 7D). Neither Aβ_25‐35_ nor UW‐MD‐95 affected the levels of insoluble Aβ_1‐42_ or soluble or insoluble Aβ_1‐40_ fractions (Figure 7E–G).

These data show both UW‐MD‐95 is protective against Aβ_25‐35_‐induced behavioral deficits, neuroinflammation, oxidative stress, apoptosis, and Aβ_1‐42_ generation in mice.

## 4 DISCUSSION

BChE is considered a pertinent target in AD. Mutations of BChE impacted the severity of AD etiology and, particularly in synergy with the ε4 allele of Apolipoprotein E, the variant K appears as a major risk factor for developing AD.^24^ BChE expression is increased in the brains of AD and Lewy bodies dementia patients, when AChE expression is markedly decreased. Moreover, these increases correlated highly significantly with the alteration of the patient's cognitive scores.^25^ BChE accelerated plaque maturation in transgenic mouse models.^6^, ^26^ Several selective BChE inhibitors have been described as anti‐amnesic and potentially neuroprotective compounds, particularly showing good ability to reduce oxidative stress and improve cell viability under glutamate stress.^27^

Research effort in medicinal chemistry to identify new and selective BChE inhibitors is currently very active.^14^, ^27^, ^28^, ^29^, ^30^ UW‐MD‐95 is the prototype of a novel series of compounds, based on a tetracyclic scaffold and a carbamate moiety, allowing potent and selective BChE inhibition.^31^ Enzymatic analyses showed that most of the carbamate‐based inhibitors exhibited a pseudo‐irreversible mechanism of inhibition, with the carbamate moiety being transferred onto the serine hydroxyl group in the catalytic center of the cholinesterase enzyme (on Ser^198^ for *h*BChE).^14^, ^19^, ^31^, ^32^, ^33^ In pharmacological studies, UW‐MD‐95 demonstrated neuroprotective effects against glutamate‐induced neurotoxicity in HT‐22 cells in vitro (with an active concentration as low as 1 μM), as well as neuroprotective effects against Aβ_25‐35_‐induced memory deficits in mice in vivo (with an active dose in the 0.3–3 mg/kg range).^14^ The compound efficacy prompted us to further examine its potential in the pharmacological Aβ_25‐35_ mouse model of AD by focusing, besides memory deficits, on neuroinflammation, oxidative stress, and Aβ species generation.

We used the model induced by ICV injection of oligomeric Aβ_25‐35_ peptide in mice as it repeatedly appeared highly pertinent to examine the neuroprotective activity of investigational drugs. Indeed, the in vivo administration of oligomeric Aβ_25–35_ in rodents resulted in alteration of APP processing toward the amyloidogenic pathway with increases of endogenous APP, C99, and Aβ_1–40/42_ levels in hippocampus and cortex and decreased expressions of Aβ clearance enzymes IDE and neprilysin.^34^, ^35^, ^36^ A strong neuroinflammatory response is induced after Aβ_25–35_ injection with astroglial and microglial reactions in several brain structures.^34^, ^37^ Pro‐inflammatory cytokines, IL‐1β, IL‐6, IL‐17, and TNFα levels are increased and anti‐inflammatory YM1/2 levels are decreased.^37^, ^38^, ^39^ Aβ_25–35_ induces oxidative stress with reactive oxygen species accumulation and lipid peroxidation in brain structures and increased iNOS levels in the hippocampus.^16^, ^40^, ^41^, ^42^ Mitochondrial alteration was shown by an increase in cytochrome c release and alteration of mitochondrial respiration.^43^ Aβ_25‐35_‐treated mice also showed alteration of long‐term potentiation and increase of long‐term depression, calcium permeability pore formation, glutamate uptake inhibition, and increase of extracellular glutamate and decreased expressions of the synaptic scaffolds PSD95, synaptophysin, and SNAP‐25 with reduction of synapses number in the hippocampus and cortex.^44^, ^45^ Cholinergic systems are specifically altered with increased expressions of AChE, decreased hippocampal ACh contents and release, decreased number of ChAT‐ and VAchT‐positive neurons, and decreased basal acetylcholine release.^13^, ^46^, ^47^ Cellular death is present with dendritic and neuronal retraction, chromatin condensation, nucleus fragmentation, mitochondrial swelling, PARP activation DNA fragmentation, increased caspase expression, and neuronal loss in the hippocampus.^38^, ^48^ Finally, this toxicity quickly results in learning and memory deficits appearing after 1 week in mice.^20^ Therefore, the Aβ_25‐35_ model allows to examine a drug effect quickly and in large cohort of animals, on a plethora of parameters descriptive of the AD pathology rendering it a model of choice to exemplify the neuroprotective potential of UW‐MD‐95 as a new neuroprotective BChE inhibitor.

We, therefore, herein reported that UW‐MD‐95 is not only neuroprotective when administered repeatedly during 1 week or even once after the Aβ_25‐35_ injection but also anti‐amnesic, when injected just before the behavioral examination in Aβ_25‐35_‐treated animals and in scopolamine‐treated mice. The drug was effective in the same dose range: 0.3–3 mg/kg with an optimal dosage of 1 mg/kg. The effect was significant in the different behavioral tests used, assessing different types of memory that are altered in both AD and the model, such as spatial and non‐spatial, short‐term and long‐term, and recognition memories. The observation that UW‐MD‐95 is effective within 30 min after a single administration suggested that the drug bioavailability is rapid and effective but also that BChE inhibition has a physiological role in neurotransmission and memory processing. Several BChE inhibitors have been previously reported to exert anti‐amnesic effects on scopolamine‐ and/or Aβ_1‐42_‐induced impairments in rodents. For instance, first, a series of carbazole derivatives showing nanomolar inhibitory potency on BChE and selectivity versus AChE were shown to attenuate scopolamine‐induced learning deficits in the water maze in rats.^29^, ^49^ Second, S06‐1011 and S06‐1031, two compounds containing a 3‐(benzimidazol‐2‐yl)‐1,2,5‐oxadiazole core, and showing a selective and sub‐micromolar inhibitory potency on BChE, attenuated scopolamine and Aβ_1‐42_‐induced memory deficits in the Morris water maze in mice.^50^ Third, sulfonamide derivatives of phenylglycine attenuated, after a 7‐day daily treatment before scopolamine, the memory deficits of rats in the Y‐maze and Barnes tests.^51^ The physiological mode of action is likely related to facilitation of the cholinergic systems for both the scopolamine and Aβ amnesia. Indeed, UW‐MD‐95 shared the ability to prevent scopolamine‐ and Aβ_25‐35_‐induced learning deficits in mice, thereby confirming a direct and rapid involvement of BChE activity in memory processes. Noteworthily, a clinical study described a very significant correlation between the rate of cognitive decline measured using the MMSE decrement per year and the activity of BChE in the temporal cortex of patients with Lewy body dementia.^25^ These observations open the way to a novel class of BChE‐based cognitive enhancers.

UW‐MD‐95 was also shown to be neuroprotective against Aβ_25‐35_‐induced learning deficits and toxicity. We observed that the neuroprotective effect of the drug was completely blocked in BChE KO animals confirming that the target is directly involved in the pharmacological effect. First, UW‐MD‐95 attenuated Aβ_25‐35_‐induced oxidative stress, by limiting lipid peroxidation, but marginally alleviated the release of cytochrome c into the cytosol, a direct index of mitochondrial membrane alteration.^52^ BChE inhibitors have been shown to elicit direct anti‐oxidant activity in cellular models, particularly after cell exposure to H_2_O_2._ ^49^ The drug may not indirectly exert its anti‐oxidant activity through specific protection of mitochondrial activity and homeostasis of the oxidative respiratory chain complex.^53^, ^54^ Second, UW‐MD‐95 attenuated Aβ_25‐35_‐induced neuroinflammation. Neuroinflammation can be seen as a consequence of amyloid toxicity and its limitation by a neuroprotective drug as a symptomatic consequence of the drug treatment. However, BChE is highly expressed in glial cells and particularly astrocytes, besides neurons in brain structures such as the amygdala, hippocampus, and thalamus.^6^, ^55^ BChE expression is regulated from glial cells and, therefore, highly involved in neuroinflammatory response. This is reported to be of major importance in amyloid plaques and neurofibrillary tangles, in AD patient brains, suggesting that glial BChE directly concours to the pathological status. Indeed, BChE activity is associated with Aβ plaques in AD and may play a role in the pathological level of Aβ species and particularly Aβ aggregation. First, a decrease in fibrillar Aβ burden was observed in 5XFAD/BChE‐KO double transgenic mice relative to 5XFAD single transgenic animals, so with a physiological level of BChE expression.^9^, ^56^ Second, BChE carries a signal sequence that promotes self‐aggregation and enhances the formation of Aβ protofibrils in vitro.^57^ Third, we observed in the Aβ_25‐35_ model an increase in soluble Aβ_1‐42_ protein that was completely prevented by the UW‐MD‐95 treatment. Although the latter model is not adequate to convincingly identify a therapeutic effect on Aβ load, contrarily to transgenic Aβ‐based mouse lines, this result is coherent with the proposed role of BChE in Aβ aggregation and accumulation.

Interestingly, the active doses of UW‐MD‐95 were within the 0.3–3 mg/kg IP range, a similar dose range as observed for the clinical references acting as ChE inhibitors.^16^ For some behavioral responses, in the passive avoidance test mainly, a bell‐shaped effect was observed, with the highest dose devoid of effect. Such hormetic effect has been observed for almost all cognitive enhancers and neuroprotective drugs,^58^ but surprisingly on most of the memory responses examined. Higher doses of UW‐MD‐95 will have to be examined in short‐term memory tests in the different paradigms used in this study to generalize the hormetic response. However, this observation will have to be taken into account for an optimal design of the drug dose regimen in case of clinical development.

Moreover, although the repeated treatment with UW‐MD‐95 was well tolerated, acute administration of the drug 30 min before the test in Aβ_25‐35_‐treated mice led to a decrease in locomotion that suggested a putative impact on the general condition of the mice. This effect was not observed in scopolamine‐treated animals that also received UW‐MD‐95 30 min the test, likely due to the fact that scopolamine enhanced locomotion per se. However, it remains that acute high‐dose effects of UW‐MD‐95 will have to be carefully examined in the future, in comparison with other BChE inhibitors.

Finally, the mode of action of BChE inhibitor appears particularly interesting as it is based on a double action on neurons, specifically ACh neurons, and on glial cells, specifically astrocytes. As the neurodegenerative process in AD is protean^5^ and affects complex interacting toxic pathways between extracellular space, neurons, and glial cells,^59^, ^60^ BChE may represent a promising target per se but also for multi‐target ligands.^61^, ^62^

## 5 CONCLUSION

We described the neuroprotective activity of UW‐MD‐95, a new carbamate‐based compound acting as a potent and selective pseudo‐irreversible BChE inhibitor, in Aβ_25‐35_‐treated mice and showed that the drug alleviated the behavioral deficits and the biochemical alterations, particularly neuroinflammation. The drug also blocked Aβ_25‐35_‐induced increase in soluble Aβ_1‐42_ content in the mouse cortex, suggesting a putative disease‐modifying effect. Further investigations in a chronic transgenic model deserve to be investigated.

## AUTHOR CONTRIBUTIONS

**Allison Carles:** Investigation, Data curation, Formal analysis, Writing, review and editing. **Matthias Hoffmann:** Investigation, Data curation, Formal analysis. **Matthias Scheiner:** Investigation, Data curation. **Lucie Crouzier:** Investigation, Data curation, Formal analysis, Writing, review and editing. **Christelle Bertrand‐Gaday:** Data curation, Writing, review and editing. **Arnaud Chatonnet:** Conceptualization, Writing, review and editing. **Michael Decker:** Conceptualization, Writing, review and editing, Funding acquisition, Project administration. **Tangui Maurice:** Formal analysis, Visualization, Writing, original draft, Conceptualization, Writing, review and editing, Funding acquisition, Project administration.

## FUNDING INFORMATION

M.D. acknowledges the Franco‐Bavarian University Cooperation Center under FK03‐2020 for travel funds. M. S. and M. H. thank the Procope Mobility Program of the French Embassy in Germany. M. D. and T. M. acknowledge support from Campus France (PHC Procope), the German Academic Exchange Service (DAAD) with funds from the Federal Ministry of Education and Research (BMBF) under project number 57702280, and the German Research Foundation (Deutsche Forschungsgemeinschaft) and the Agence Nationale de la recherche (ANR) under DFG DE 1536/12‐1 and ANR 22‐CE92‐0080 for funding.

## CONFLICT OF INTEREST STATEMENT

The authors declare that they have no conflict of interest.

## Supporting information


Figure S1.

Figure S2.

Figure S3.

Appendix S1.

## Data Availability

The data that support the findings of this study are available from the corresponding author upon reasonable request.
