# Supplementary figures and images for: The selective butyrylcholinesterase inhibitor UW‐MD‐95 shows symptomatic and neuroprotective effects in a pharmacological mouse model of Alzheimer's disease
Source: CNS Neurosci Ther. 2024 Jun 17;30(6):e14814. doi: 10.1111/cns.14814 (PMC11183908; doi:10.1111/cns.14814)

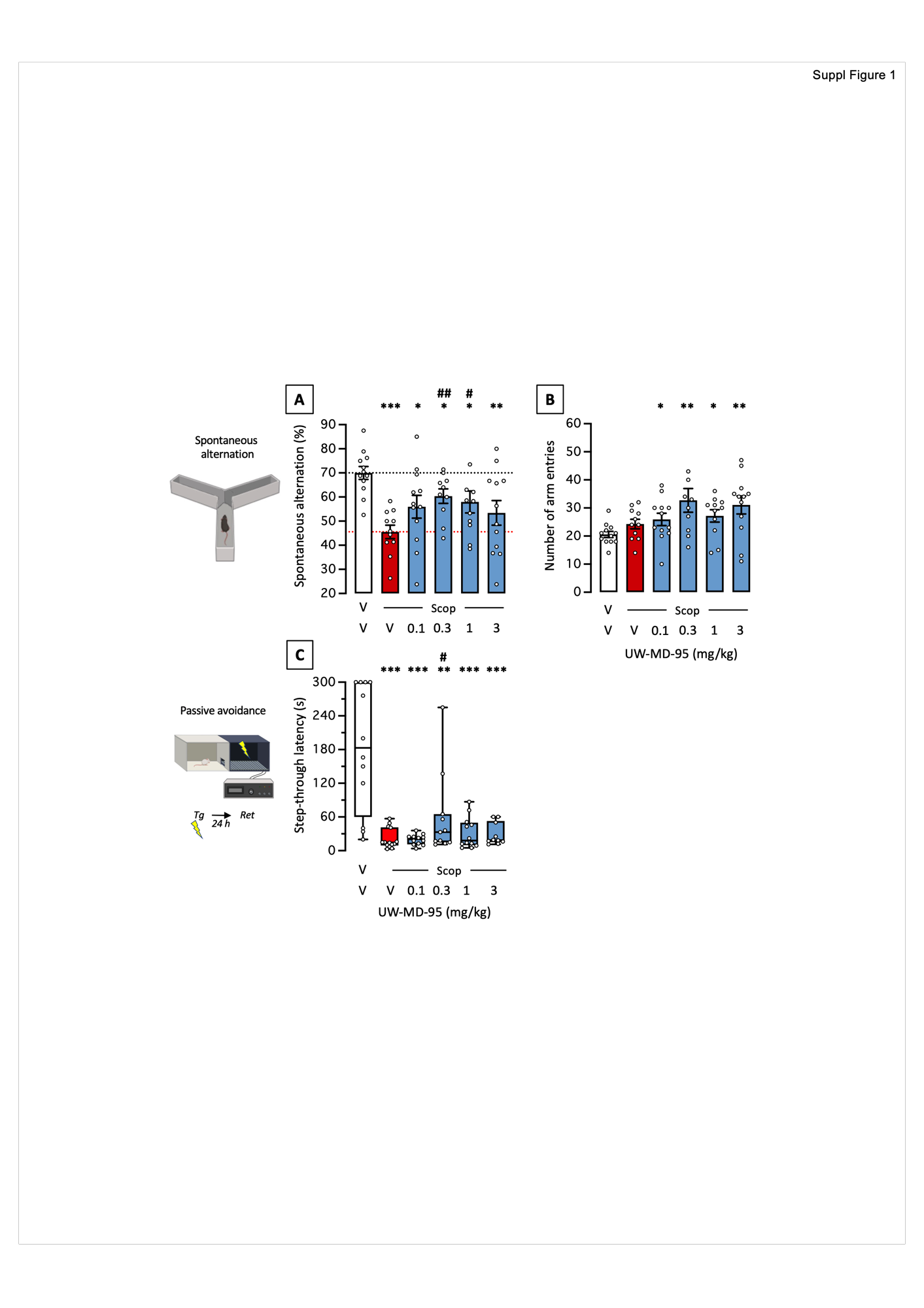

Supplement: Supplementary file 1 — Figure S1. [file CNS-30-e14814-s004.tif]

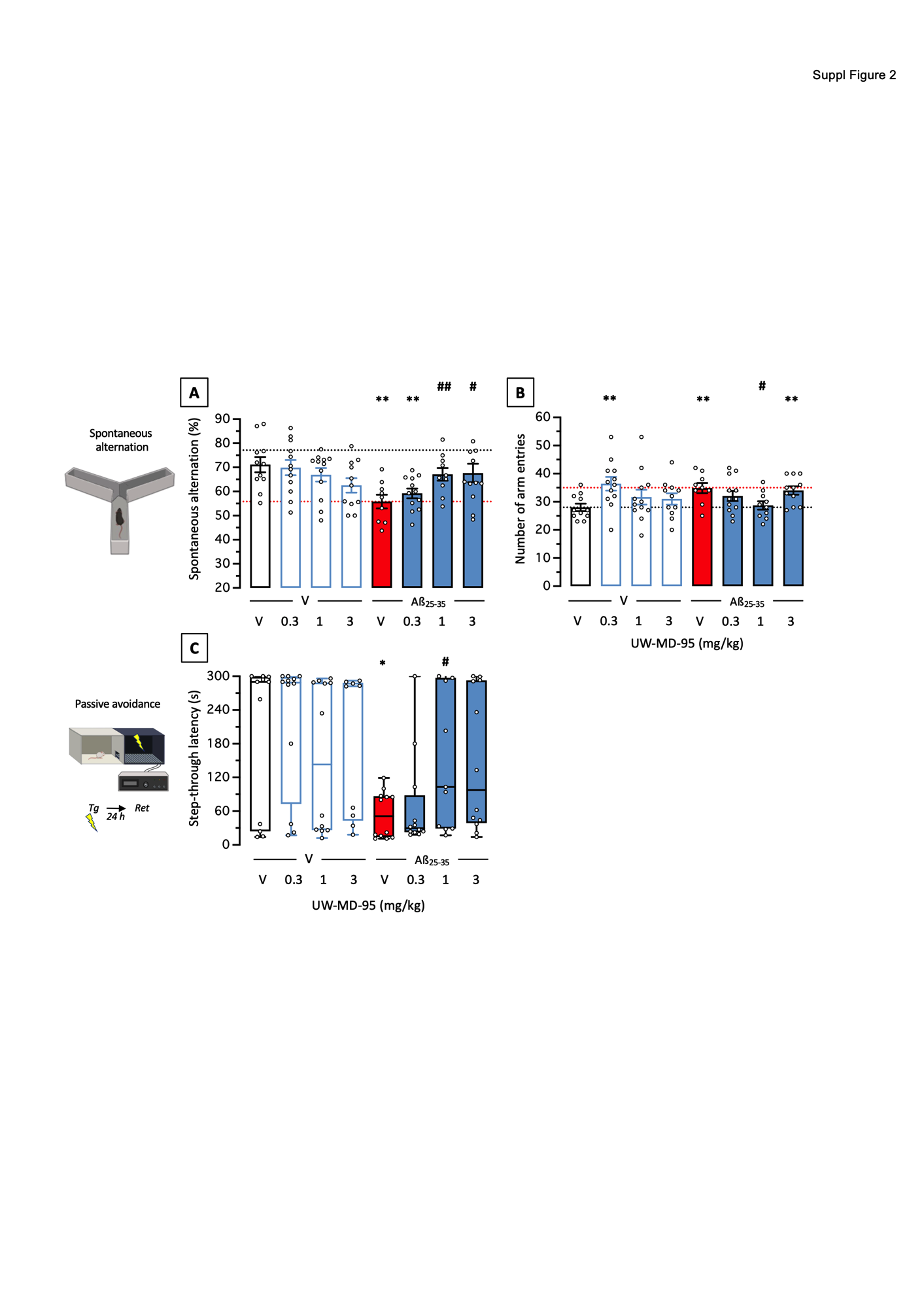

Supplement: Supplementary file 2 — Figure S2. [file CNS-30-e14814-s002.tif]

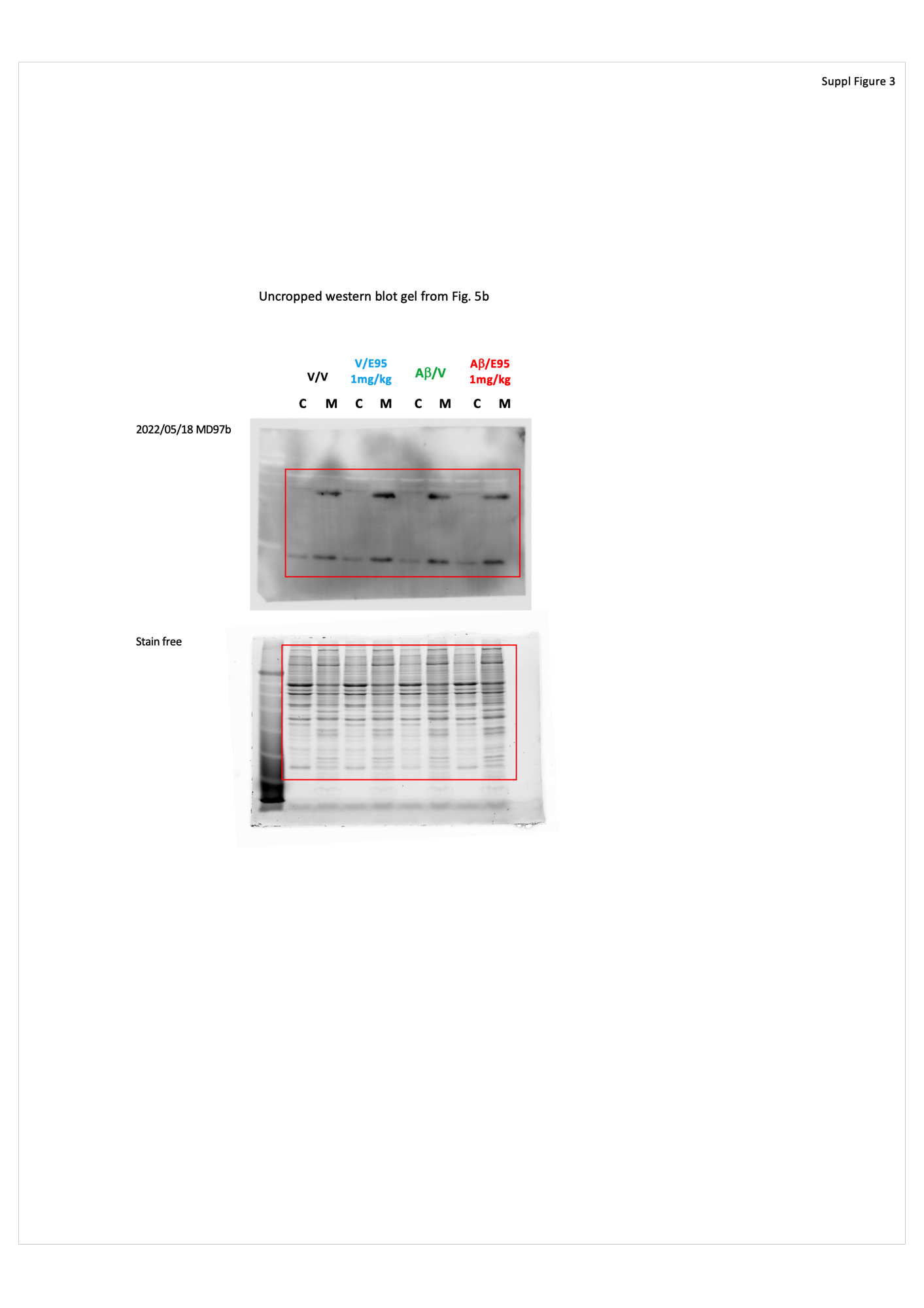

Supplement: Supplementary file 3 — Figure S3. [file CNS-30-e14814-s003.tif]
