# Supplementary material for: The selective butyrylcholinesterase inhibitor UW‐MD‐95 shows symptomatic and neuroprotective effects in a pharmacological mouse model of Alzheimer's disease
Source: CNS Neurosci Ther. 2024 Jun 17;30(6):e14814. doi: 10.1111/cns.14814 (PMC11183908; doi:10.1111/cns.14814)
Supplement: Supplementary file 4 — Appendix S1. [file CNS-30-e14814-s001.docx]

**Supplementary Table 1.** Commercial kits used in the study.

*Marker Supplier Reference Batch no.*

Bax Cloud-Clone Corp SEB343MU 4FBE513B6F

Bcl-2 Cloud-Clone Corp SEA778MU 089D7E6339

GFAP Cloud-Clone Corp SEA068MU 0F5E05AE12

Iba-1 Cloud-Clone Corp SEC288MU 405293E2FE

IL-6 Cloud-Clone Corp SEA079MU 72431BCFFC

TNFα Cloud-Clone Corp SEA133MU 2F9E677166

Aβ_1-40_ Thermo Fisher KMB3481 322057-004

Aβ1-42 Thermo Fisher KMB3441 335204-003

*Abbreviations:* Bax, bcl-2-like protein 4; Bcl-2, B-cell lymphoma 2, GFAP, glial fibrillary acidic protein; Iba-1, ionized calcium-binding adaptor molecule 1; IL-6, interleukin-6; TNFα, tumor necrosis factor-α.

**Supplementary Table 2.** Complete Statistical calculations.

**Figure 1**

1D one-way ANOVA: F_(4,137)_ = 0.7216, p = 0.5786

one-sample t-test (*vs*. 0): t = 7.058, df = 26, p < 0.0001 *** for V/V

t = 7.962, df = 26, p < 0.0001 *** for Aß/V

t = 8.677, df = 29, p < 0.0001 *** for Aß/0.3

t = 8.471, df = 29, p < 0.0001 *** for Aß/1

t = 8.419, df = 27, p < 0.0001 *** for Aß/3

1G one-way ANOVA: F_(4,52)_ = 1.162, p = 0.3382

one-sample t-test (*vs*. 0): t = 1.758, df = 12, p = 0.1042 for V/V

t = 2.449, df = 13, p = 0.0293 * for Aß/V

t = 4.773, df = 9, p = 0.0010 ** for Aß/0.3

t = 2.386, df = 9, p = 0.0408 * for Aß/1

t = 2.884, df = 9, p = 0.0181 * for Aß/3

1I one-way ANOVA: F_(4,52)_ = 2,205, p = 0.08112

one-sample t-test (*vs*. 0): t = 1.145, df = 11, p = 0.2767 for V/V

t = 1.641, df = 12, p = 0.1267 for Aß/V

t = 3.088, df = 9, p = 0.7645 for Aß/0.3

t = 3.221, df = 9, p =0.0105 * for Aß/1

t = 0.5559, df = 11, p = 0.5888 for Aß/3

**Figure 2**

2A one-way ANOVA: F_(4,52)_ = 5.341, p = 0.0195 *

2B one-way ANOVA: F_(4,52)_ = 9.790, p < 0.0001 ***

2C one-way ANOVA: F_(4,52)_ = 0.9784, p = 0.4274

one-sample t-test (*vs*. 50%): t = 1.248, df = 13, p = 0.2339 for V/V

t = 0.1088, df = 10, p = 0.9155 for Aß/V

t = 1.707, df = 11, p = 0.1158 for Aß/0.3

t = 0.4468, df = 10, p = 0.6645 for Aß/1

t = 0.04093, df = 8, p = 0.9684 for Aß/3

2D one-way ANOVA: F_(4,52)_ = 10.80, p < 0.0001 ***

one-sample t-test (*vs*. 50%): t = 14.30, df = 13, p < 0.0001 *** for V/V

t = 3.995, df = 10, p = 0.0025 ** for Aß/V

t = 11.37, df = 11, p < 0.0001 *** for Aß/0.3

t = 14.44, df = 10, p < 0.0001 *** for Aß/1

t = 10.89, df = 8, p < 0.0001 *** for Aß/3

2E Kruskal-Wallis ANOVA: H = 18.88, p = 0.0008 ***

**Figure 3**

3A one-way ANOVA: F_(4,92)_ = 2.550, p = 0.0444 *

3B one-way ANOVA: F_(4,92)_ = 0.7675, p = 0.5491

3C one-way ANOVA: F_(4,54)_ = 0.3307, p = 0.8561

one-sample t-test (*vs*. 50%): t = 0.6170, df = 11, p = 0.5498

t = 0.8065, df = 10, p = 0.4387

t = 1.109, df = 11, p = 0.2909

t = 0.4820, df = 11, p = 0.6393

t = 0.4823, df = 11, p = 0.6390

3D one-way ANOVA: F_(4,54)_ = 2.567, p = 0.0483 *

one sample t-test (*vs*. 50%): t = 4.778, df =11, p = 0.0006 *** for V/V

t = 0.2908, df = 10, p = 0.7772 for Aß/V

t = 0.6799, df = 11, p = 0.5106 for Aß/0.3 t = 2.566, df = 11, p = 0.0262 for Aß/1

t = 0.1874, df = 11, p = 0.8547 for Aß/3

3E Kruskal-Wallis ANOVA: H = 9.889, p = 0.0423 *

**Figure 4**

4A one-way ANOVA: F_(4,45)_ = 4.529, p = 0.0037 **

4B one-way ANOVA: F_(4,40)_ = 3.07, p = 0.0269 *

4C Kruskal-Wallis ANOVA: H = 10.83, p = 0.029 *

4D Kruskal-Wallis ANOVA: H = 20.3, p = 0.0004 ***

**Figure 5**

5A one-way ANOVA: F_(4,42)_ = 4.204, p = 0.0059 **

5B Two-way ANOVA: F_(1,33)_ = 4.485, p < 0.0418 *, for the Aβ_25-35_ treatment

F_(1,33)_ = 0.1795, p = 0.1895, for the UW-MD-95 treatment

F_(1,33)_ = 1.577, p = 0.2180, for the interaction

5C one-way ANOVA: F_(4,31)_ = 3.598, p = 0.0160 *

5D one-way ANOVA: F_(4,28)_ = 2.897, p = 0.0400 *

5E one-way ANOVA: F_(4,29)_ = 6.179, p = 0.0010 **

5F one-way ANOVA: F_(4,29)_ = 2.792, p = 0.0447 *

**Figure 6**

6C Two-way ANOVA: F_(1,18)_ = 6.236, p < 0.0224 *, for the Aβ_25-35_ treatment

F_(1,18)_ = 0.09463, p = 0.7619, for the UW-MD-95 treatment

F_(1,18)_ = 8.152, p = 0.0105 *, for the interaction

6D Two-way ANOVA: F_(1,18)_ = 4.510, p = 0.0478 *, for the Aβ_25-35_ treatment

F_(1,18)_ = 1.556, p = 0.2282, for the UW-MD-95 treatment

F_(1,18)_ = 12.16, p = 0.0026 **, for the interaction

6F Two-way ANOVA: F_(1,19)_ = 21.64, p = 0.0002 ***, for the Aβ_25-35_ treatment

F_(1,19)_ = 0.9132, p = 0.3513, for the UW-MD-95 treatment

F_(1,19)_ = 9.092, p = 0.0071 **, for the interaction

6G Two-way ANOVA: F_(1,19)_ = 9.195, p = 0.0069 **, for the Aβ_25-35_ treatment

F_(1,19)_ = 0.0887, p = 0.7691, for the UW-MD-95 treatment

F_(1,19)_ = 1.58, p = 0.2240, for the interaction

6I Two-way ANOVA: F_(1,20)_ = 2.725, p = 0.1144, for the Aβ_25-35_ treatment

F_(1,20)_ = 2.253, p = 0.1490, for the UW-MD-95 treatment

F_(1,20)_ = 0.1529, p = 0.6999, for the interaction

6J Two-way ANOVA: F_(1,19)_ = 1.258, p = 0.2761, for the Aβ_25-35_ treatment

F_(1,19)_ = 0.2327, p = 0.6350, for the UW-MD-95 treatment

F_(1,19)_ = 7.931, p = 0.0110 *, for the interaction

**Figure 7**

7A one-way ANOVA: F_(4,32)_ = 3.027, p = 0.0318 *

7B one-way ANOVA: F_(4,31)_ = 0.6994, p = 0.5982

7C one-way ANOVA: F_(4,29)_ = 2.197, p = 0.0942

7D Two-way ANOVA: F_(1,20)_ = 37.05, p < 0.0001 ***, for the Aβ_25-35_ treatment

F_(1,20)_ = 67.47, p < 0.0001 ***, for the UW-MD-95 treatment

F_(1,20)_ = 38.13, p < 0.0001 ***, for the interaction

7E Two-way ANOVA: F_(1,20)_ = 0.3683, p = 0.5508, for the Aβ_25-35_ treatment

F_(1,20)_ = 0.1113, p = 0.7422, for the UW-MD-95 treatment

F_(1,20)_ = 2.431, p = 0.1346, for the interaction

7F Two-way ANOVA: F_(1,20)_ = 0.1147, p = 0.7383, for the Aβ_25-35_ treatment

F_(1,20)_ = 0.0211, p = 0.8860, for the UW-MD-95 treatment

F_(1,20)_ = 0.8454, p = 0.3688, for the interaction

7G Two-way ANOVA: F_(1,20)_ = 0.2055, p = 0.6552, for the Aβ_25-35_ treatment

F_(1,20)_ = 2.007, p = 0.1719, for the UW-MD-95 treatment

F_(1,20)_ = 2.007, p = 0.1719, for the interaction

**Supplementary Figure 1**

S1A one-way ANOVA: F_(5,62)_ = 4.109, p = 0.0028 **

S1B one-way ANOVA: F_(5,62)_ = 2.999, p = 0.0173 *

S1C Kruskal-Wallis ANOVA: H = 23.74, p = 0.0002 ***

**Supplementary Figure 2**

S2A one-way ANOVA: F_(7,80)_ = 3.139, p = 0.0056 **

S2B one-way ANOVA: F_(7,80)_ = 2.174, p = 0.0452 *

S2C Kruskal-Wallis ANOVA: H = 14.69, p = 0.0402 *
